# Orthodontic Compliance Assessment: A Systematic Review

**DOI:** 10.1016/j.identj.2022.07.004

**Published:** 2022-08-10

**Authors:** Marek Nahajowski, Joanna Lis, Michał Sarul

**Affiliations:** a Department of Integrated Dentistry, Wroclaw Medical University, Wroclaw, Poland; b Department of Orthodontics and Dentofacial Orthopedics, Wroclaw Medical University, Wroclaw, Poland

**Keywords:** Compliance, Microsensors, Orthodontics, Removable appliances, Malocclusion

## Abstract

**Objectives:**

The aim of this review was to determine whether the type of removable appliance, as well as the age and sex of the patient, may affect the extension or reduction of wear time by assessing the correlation between the mean actual and orthodontist-recommended wear times.

**Methods:**

Randomised case control trials, cohort studies, case series, observational studies, reviews, and retrospective analyses were identified. The quality of the studies was assessed using the Cochrane Collaboration Tool and modified Newcastle-Ottawa Scale. The electronic databases Embase, PubMed, Scopus, and Web of Science were reviewed, and 542 articles were obtained, of which 31 were qualified for qualitative synthesis. The data from 1674 participants were collected and a weighted average was determined for the mean wear time of each appliance.

**Results:**

Regardless of the type of extra- or intraoral appliances, mean wear time was shorter than recommended, although patients using intraoral appliances cooperated more. The best compliance was noted for Schwarz appliances (73.70%) and plate retainers (85%). There was no evidence of an influence of patients’ age and sex on compliance during treatment.

**Conclusions:**

The considerable inconsistency and imprecision of articles could affect the reliability of the results. Previous studies analysing the effectiveness of treatment with removable appliances based on an arbitrarily assumed average wear time need to be revised in order to verify the actual wear time with the use of microsensors.

## Introduction

Removable orthodontic appliances are commonly used in the treatment of malocclusion, especially in 7- to 25-year-old patients in developmental age.^1^, ^2^, ^3^ Such appliances carry a low risk of complications and, above all, are inexpensive and ideal for solving many problems in early and interceptive orthodontic treatment and thus in the general treatment of children and adolescents.^1^^,^^4^ However, the most important factor in achieving the desired effect with removable appliances is a patient's cooperation, which involves, amongst other things, compliance with the recommended daily wear time (DWT), which according to many researchers is often not observed.^5^, ^6^, ^7^, ^8^, ^9^

Objective and efficient DWT measurement is presently ensured by microsensors fitted in removable appliances, both intra- and extraoral^10^, ^11^, ^12^ ones. With the use of such sensors, it has already been proven that patients do not comply with the recommended DWT and usually shorten it arbitrarily.^13^ Nevertheless, the question of which specific factors are significantly correlated with better or worse patient compliance remains open. Therefore, the aim of our systematic review was to determine whether the type of removable appliance, as well as age and sex of the patient, might result in extending or shortening of DWT.

## Materials and methods

The systematic review was conducted according to the Preferred Reporting Items for Systematic Reviews and Meta-Analyses protocol (PRISMA^14^) and registered in PROSPERO under the number CRD42021243067. The data sets used and/or analysed in the presented study are available from the corresponding author on reasonable request.

### Eligibility criteria

The main research question and the inclusion criteria were defined in the PI(E)COS (Population, Intervention (Exposure), Comparison, Outcome) format.

• P: 7- to 25-year-old patients without systemic diseases treated with any type of removable appliance with microsensors or other devices measuring DWT
• I: Patients wearing removable appliances for the time recommended by the orthodontist
• C: Patients not wearing removable appliances for the time recommended by the orthodontist
• O: Compliance levels in relation to stipulated levels of wearing the appliances; secondary outcomes: factors influencing compliance levels (type of the appliance, age, sex)

Regarding the type of articles, only randomised case control trials, cohort studies, case series, observational studies, and retrospective analyses were eligible. Case reports, letters to the editor, studies with fixed appliances, and studies with several types of removable appliances, in which the size of individual groups was not specified, were excluded.

The study was designed to verify whether the type of appliance as well as the sex and age of the patient had any effect on compliance, that is, the actual DWT (aDWT) defined as the correlation of mean DWT and DWT recommended by the doctor (aDWT = mean DWT / recommended DWT x 100).

### Information sources and search strategy

The electronic databases Embase, PubMed, Scopus, and Web of Science were reviewed to obtain articles specified in the methodology. The search included all publications available up to April 2022 and did not include any additional criteria for language or publication date. We also performed hand searching, including the primary sources available in the reviews, doctoral theses, and unpublished research known to the authors. In order to include more grey literature in our systematic review, we contacted the producers of microsensors used in the studies we qualified in order to obtain information about unpublished works on the issue analysed by us. The search strategy is shown in Appendix Table 1.

### Selection process

The literature search and assessment of relevance were performed independently by 2 authors (MN and MS). The final list of qualified articles was established on the basis of a vote in which all authors took part. The works accepted by all authors were qualified for further analysis.

### Data collection process

Data extraction was performed independently by 2 authors (MN and MS). The original investigators were contacted for missing information. All authors discussed disagreements until consensus was reached.

### Data items

From each article, we extracted year of publication, type of article, number and sex of patients, average patient age, types of orthodontic appliances used in the study, number of patients treated with a given device, type of microsensor measuring the wear time, recommended and actual wear time, and duration of the study (including dropouts).

None of the studies found accurate information on the sex and age of patients in individual groups (only this information was provided before the classification of patients into groups), whilst in our analysis we used the ready-made data on the correlation between the wear time and the age and sex of patients collected from individual articles. Data extracted from papers are presented in Table 1.

**Table 1:** Characteristics of the analysed studies.

No	Author, year	Article type	Number and sex of patients	Mean age (y)	Group 1 (appliance)	Group 2 (appliance)	Group 3 (appliance)	Type of microsensor	Stipulated wear time	Mean DWT	Study duration
**1**	Arponen, 2020^17^	Prospective cohort study	30	13.8 ± 4.7; range: 12-18	HG: 10	TB: 20		TheraMon®	12 h/d HG, 18 h/d TB	HG: 5 h/d, TB: 7.5 h/d	7-23 mo (13 mo average); 390 d (210-690 d) with follow-ups each 90 d; 17 dropouts
**2**	Parekh, 2019^37^	RCT	55 (25 M, 37 F)	12.37 ± 0.95; range: 10-14	TB (FT): 25	TB (PT): 30		TheraMon®	12 h/d PT, 22 h/d FT	8.78 ± 3.77 h/d PT, 12.38 ± 5.89 h/d FT	12 mo = 365 d (follow-ups each 45-60 d); 7 dropouts
**3**	Charavet, 2018^40^	Prospective cohort study	69 (37 M, 32 F)	7.8 ± 1.1	Planas functional appliance			TheraMon®	24 h/d	15.8 ± 5.2 h/d	9 mo; 270 d (follow-up each 90 d); 10 dropouts
**4**	Brandao, 2006^29^	Prospective cohort study	21 (10 M, 11 F)	14.8; range: 11-19.5	HG (T1: uninformed of measurement; T2: informed of measurement)			Compliance Science System and Affirm Smart Headgear Modules, Ortho Kinetics	14 h/d	5.6 ± 4.4 h/d T1; 7.0 ± 5.4 h/d T2	First 3 mo of HG wear
**5**	Agar, 2005^28^	Prospective cohort study	51 (17 M, 34 F)	12.92	HG (G1: compliant patients,(23) G2: noncompliant patients, 28)			Compliance Science System and Affirm Smart Headgear Modules, Ortho Kinetics	16 h/d	G1: 18.34 h/d; G2: 9.10 h/d	6 mo
**6**	Cureton, 1993^18^	Prospective cohort study	28 (10 M, 18 F)	<10	HG			Fabricated from commercial wristwatch	12 h/d	6.5 ± 3.5 h/d	Up to 3 mo, with at least 3 mo of HG wear
**7**	Cureton, 1993^20^	Prospective cohort study	28 (10 M, 18 F)	<10	HG			Fabricated from commercial wristwatch	12 h/d	7.9 h/d with calendar; 5.3 h/d without calendar	Up to 3 mo, with at least 3 mo of HG wear
**8**	Ghislanzoni Huanca, 2019^33^	Prospective cohort study	20 (9 M, 11 F)	10.2 ± 1.2; range: 8-12	HG			Smartgear, Swissorthodontics AG	12 h/d	8.7 h/d only days worn; 6.4 h/d including all treatment period	8-9 mo; 249 ± 15 d
**9**	Arreghini, 2016^31^	Prospective cohort study	30 (16 M, 14 F)	Class II (n = 14): 9.8; class III (n = 16): 10.0; range: 6-15	Frankel functional appliance: 11	Facemask: 16	Bionator functional appliance: 3	TheraMon®	13 h/d	Frankel: 8.6 ± 1.9; Bionator: 12.6 ± 1.8; facemask: 8.0 ± 3.2	8 mo (range: 2-16 mo); 240 d with follow-ups each 30 d (range: 60-480 d)
**10**	Schott, 2017^32^	Prospective cohort study	109 (54 M, 55 F)	12.3 ± 2.9; range: 6-20	Schwarz plates: 33	Functional appliances: 34	Retention plates: 42	TheraMon®	15.1 ± 0.9h/d (plates); 15.2 ± 0.5 h/d (functional); 13.4 ± 2.7 h/d (retainers)	11.9 ± 3.0 h/d (plates); 10.2 ± 3.3 h/d (functional); 9.0 ± 4.9 h/d (retainers)	12 mo (first follow-up appointment); EP: 63.4 ± 36.8 d; RFA: 59.1 ± 26.2 d; R: 102.8 ± 65.8 d
**11**	Tsomos, 2014	Cross-sectional cohort study	45 (25 M, 20 F)	Retention: 12.7; range: 7.2-21.5; functional appliances: 11.8 (range: 8.0-15.8)	Functional appliances (Frankel II/III, Sander II, cow catch): 14	Hawley and Essix retainers (retention plates): 31		TheraMon®	G1: 14 h/d (for functional); G2: 8 h/d (for retainers)	9 h/d	186 d (55-318 d); R: 177 d (range: 55-293 d); RFA: 186 d (range: 96-318 d); 4 dropouts
**12**	Schaefer, 2014	Prospective cohort study	141 (88 M, 53 F)	10.95 ± 1.87; range: 7-15	Standard activator or class III activator: functional appliances: 71	Schwarz plates: 70		TheraMon®	15 h/d	Functional: 9.5 h/d; plate: 10.1 h/d; overall: 9.7 h/d	First 3 mo of appliance wear; ≥90 d with follow-ups each 100 d
**13**	Schott, 2014	Prospective cohort study	(56 M, 36 F); normal weight: 53 (32 M, 21 F); overweight: 39 (24 M, 15 F)	G1: 11.2 ± 2.2; G2: 11.2 ± 2.6; range: 7.9-15.9	Schwarz plates: 42	Jumping-the-bite appliance functional appliance: 53		TheraMon®	11 h/d (9 h/d)	Normal weight: 9.3 h/d; overweight: 9.2 h/d	5 mo, 150 d; 3 dropouts
**14**	von Bremen, 2018^16^	Prospective cohort study	114 (undefined sex); normal weight: 57; overweight: 57	G1 (plate): 10.4 ± 2.08; G2: 11.6 ± 3.22 (Sander II)	Schwarz plates: (normal weight: 25; overweight: 25)	Sander II functional appliances: (normal weight: 32; overweight: 32)		TheraMon®	15 h/d	Schwarz plate: 9.6 ± 2.5 h/d; Sander II: 8.7 ± 2.4 h/d	6 mo (180 d)
**15**	Trakyali, 2008^30^	Prospective cohort study	30 (16 M, 14 F)	IG: 10.78 ± 1.06; CG: 10.07 ± 1.09	HG cervical with hypnosis (IG)	HG cervical without hypnosis (CG)		Compliance Science System and Affirm Smart Headgear Modules, Ortho Kinetics	16 h/d	IG: 3 mo: 13.75 ± 5.29; 6 mo: 12.13 ± 4.49; CG: 3 mo: 8.92 ± 3.41; 6 mo: 9.68 ± 4.43	Up to 6 mo
**16**	Cole, 2002^27^	Prospective cohort study	16 (8 M, 8 F)	12.7	HG			Compliance Science System and Affirm Smart Headgear Modules, Ortho Kinetics	10-12 h/d	6.78 h/d	Up to 6 mo, with at least 3 mo of HG wear
**17**	Clemmer, 1979^23^	Prospective cohort study	20 (11 M, 9 F)	13.8; range: 11-17	HG			Aledyne Timer, Aledyne Co.	12-14 h/d	7.43 h/d	Up to 6-9 wk
**18**	Bartsch, 1993^26^	Prospective cohort study	77 (40 M, 37 F)	10.2 ± 1.51	Bionator functional appliance			Fabricated from commercial wristwatch	15 h/d	8.7 h/d	3.9 mo
**19**	Hyun, 2015^35^	RCT (crossover study)	18 (7 M, 11 F); IG: 8 (3 M, 5 F); CG: 10 (4 H, 6 M)	15.44 ± 1.38; IG: 15.13 ± 1.55; CG: 15.70 ± 1.25; range: 14-17.6	Maxillary Hawley retainer modified: informed about sensors (IG)	Maxillary Hawley retainer modified informed about sensors (CG)		SMART Retainer Microsensor	19 h/d	IG: T1: 16.3 ± 4.39; T2: 15.6 ± 4.77; CG: T1: 10.6 ± 5.36; T2: 11.1 ± 6.08	12 wk, 90 d with follow-ups at 42 d and at 84 d; 4 dropouts
**20**	Bos, 2007^21^	Prospective cohort study	56 (19 M, 37 F)	12.89 ± 2.16; range: 10-22	HG			Termochron i-Button	12 h/d	5.58 ± 4.39 h/d	Up to 29 d; duration of treatment: 30-1140 d (mean: 240 d)
**21**	Schott, 2014^22^	Prospective cohort study	28 (16 M, 12 F)	10.6 ± 2.2; range: 7.7-17.4	Schwarz plates			TheraMon®	15 h/d	T1 (55 ± 11 d): n = 28, 12.8 ± 3.8; T2 (57 ± 13 d): n = 26, 13.3 ± 3.9; T3 (58 ± 7 d): n =13, 12.7 ± 4.8	First 6 mo; at 55 ± 11 days after treatment begins; at 57 ± 13 d after first follow-up; at 180 d
**22**	Ackerman, 2011	RCT	19 (10 M, 13 F)	15.4	Maxillary Hawley retainer			Smart Retainer (Georgia)	20 h/d	17.5 ± 16.9 h/d	30 d; 4 dropouts
**23**	Al.-Moghrabi, 2019^34^	RCT	62 (42 M, 42 F)	17.23 ± 1.9; range: 12-21	Vacuum formed retainer (IG: with reminder app, CG: without reminder app)			TheraMon®	22 h/d	IG: 7.25 ± 6.71 h/d; CG: 6.21 ± 7.86 h/d	365 d with follow-ups at 90 d; 22 dropouts
**24**	Vagdouti, 2019^36^	RCT	76 (36 M, 40 F)	14.8 ± 1.5; range: 12-18	Hawley retainer: 35	Vacuum formed retainer: 42		TheraMon®	24 h/d	Hawley retainer: 15.3 ± 6.8 h/d; vacuum formed retainer: 18.3 ± 4.6 h/d	90 d with follow-ups at 30 d; 1 dropout
**25**	Schott et al, 2013^6^	Prospective cohort study	100 (52 M, 48 F)	15.46; range: 13-20	Bimaxillary Hawley retainer: 71; functional appliance retainer: 29			TheraMon®	≥8 h	7 h/d	450 d with follow-ups each 100 d
**26**	Sarul et al, 2019^24^	Prospective cohort study	97 (51 M, 46 F)	9-12	Schwarz plates: 56	TB functional appliance: 41		TheraMon®	≥12-14 h	Active plates: 7.88 ± 3.49 h/d; TB: 7.37 ± 2.76 h/d	270 d with follow-ups each 45 d
**27**	Kawala et al, 2013^41^	Prospective cohort study	41 (20 M, 25 F)	9.2 (8.9 M/10.6 F)	Schwarz plate			TheraMon®	9 h	8.3 ± 0.4 h/d	75 d (30-120 d); follow-ups each 30 d; 4 dropouts
**28**	Al.-Kurwi et al, 2016^38^	Prospective cohort study	28 (20 M, 8 F)	11.6 ± 1.25	van Beek activator functional appliance			TheraMon®	12 h	7.75 ± 3.66 h/d	First 3 follow-ups (between 108 and 279 d); 10 dropouts
**29**	Zinad et al, 2017^39^	Prospective cohort study	88 (47 M, 41 F)	12.6 ± 1.21	RFA (monoblock type) functional appliance			TheraMon®	16 h	10.2 ± 2.86 h/d	≥180 d (follow-ups each 45-60 d); 10 dropouts
**30**	Kutay et al, 2021^19^	Prospective cohort study	30 (16 M, 14 F)	MB: 12.73 ± 1.38; TB: 12.27 ± 0.96	MB	TB		TheraMon®	15 h/d	MB: 11.02 ± 4.40 h/d; TB: 10.33 ± 3.51 h/d	6 mo; follow-ups each 4 wk; no dropouts
**31**	Sarul et al, 2021^13^	Prospective cohort study	55 (26 M, 29 F)	10.4	TB			TheraMon®	12 h/d	7.60 ± 3.12 h/d	18 mo; follow-ups each 4-6 wk; 14 dropouts

DWT, daily wear time; RCT, randomised control trial; M, males; F, females; HG, headgear; TB, twin-block; PT, part-time; FT, full-time; MB, monoblock; IG, intervention group; CG, control group; RFA, monoblock type functional appliance; EP, Schwarz plate type appliance; R, retention appliance.

### Qualitative analysis and bias analysis

The quality assessment of the articles was carried out independently by 2 researchers (MN, MS). The following criteria were used for assessing the risk of bias in randomised control trials (RCTs): random sequence generation, allocation concealment, blinding of participants and personnel, blinding of assessors, incomplete outcome data, selective reporting of outcomes, and other potential sources of bias, based on the guidelines for the Cochrane Collaboration Tool. The quality of the Controlled Clinical Trials was assessed according to the modified Newcastle-Ottawa Scale (NOS).^15^

### Statistical analysis

Due to the heterogeneity of articles included in the review, it was not possible to perform a meta-analysis. In order to compare figures, a weighted average was determined for the mean DWT in relation to the size of study groups, taking into account different criteria for classification into each group.

## Results

### Study selection

A total of 542 articles were obtained. After removing duplicates, 50 studies remained and were carefully analysed. Subsequently, after reading full texts of the obtained articles and excluding a further 19 studies that were considered not to comply with the inclusion criteria for the review (Appendix Table 2), 31 articles were eligible for final analysis. As many as 26 of those were prospective cohort studies and 5 were randomised clinical trials. The selection process diagram is shown in the flowchart (Appendix Figure 1).

### Research characteristics

The group size in our study ranged from a minimum of 19^12^ to a maximum of 141 patients.^8^ Sex of the participants was reported in most studies, with the exception of 2 articles.^16^^,^^17^ The minimum observation period was 30 days,^12^ and the maximum period was 23 months.^17^ Loss of patients during the study ranged from 0^8^^,^^16^^,^^18^, ^19^, ^20^, ^21^, ^22^, ^23^, ^24^, ^25^, ^26^, ^27^, ^28^, ^29^, ^30^, ^31^, ^32^, ^33^ to a maximum of 22.^34^

Retainers,^7^^,^^12^^,^^25^^,^^34^, ^35^, ^36^ functional appliances,^7^, ^8^, ^9^^,^^13^^,^^16^^,^^17^^,^^19^^,^^24^^,^^26^^,^^31^^,^^37^, ^38^, ^39^, ^40^ active removable appliances,^8^^,^^9^^,^^16^^,^^22^^,^^24^^,^^32^^,^^41^ and extraoral appliances^17^^,^^18^^,^^20^^,^^21^^,^^23^^,^^27^, ^28^, ^29^, ^30^, ^31^^,^^33^ were used in the study. Objective measurement of DWT was carried out primarily using TheraMon® microsensors. In addition, Smart Retainer®,^12^^,^^35^ Thermochron i-Button®,^21^ Smartgear®,^33^ Compliance Science System and Affirm Smart Headgear Modules®,^27^, ^28^, ^29^, ^30^ Aledyne Timer®,^23^ and modified wristwatches^18^^,^^20^^,^^26^ were used. The recommended DWT ranged from 8^25^ to 24 hours per day^36^^,^^40^ and thus varied according to the type of orthodontic treatment (Table 1).

### Risk of bias in studies

The results of the Cochrane analysis of RCTs eligible for review proved their low risk of bias (Appendix Table 3).

The quality of case control and cohort studies as assessed by NOS was high. As many as 17 eligible studies^7^^,^^8^^,^^30^, ^31^, ^32^, ^33^^,^^38^^,^^39^^,^^41^^,^^9^^,^^13^^,^^18^^,^^19^^,^^26^, ^27^, ^28^, ^29^ scored 8 out of total 9 points, whilst the remaining studies^6^^,^^16^^,^^17^^,^^20^, ^21^, ^22^, ^23^, ^24^^,^^40^ scored 7 out of 9 points. The results of those analyses are presented in Appendix Tables 3 and 4.

### Results of individual studies and syntheses

#### Recommended DWT vs mean DWT

The mean DWT of removable appliances included in the study, measured objectively with microsensors, was shorter than the time recommended by orthodontist (Table 1).

#### DWT vs appliance type

Statistically significant differences in compliance with orthodontist recommendations regarding DWT depending on the type of removable appliances were observed only in 2 studies.^7^^,^^39^

As was stated in the methodology section, in our systematic review—in addition to providing mean DWT—aDWT was determined based on a weighted average for each type of appliance included in the papers, taking into account the size of study groups (Table 2).

**Table 2:** Mean DWT and aDWT related to the removable appliance types.

	Extraoral appliances (n = 317)		Intraoral appliances (n = 1404)														
	HG	FM	SP	Removable functional appliances (n = 713)										Retainers (n = 371)			
				**TB**	**PL**	**FR**	**BIO**	**SA**	**ACT**	**VBA**	**MB**	**FR, SA, CC (UN)**^1^	**Or (UN)**^2^	**HR**	**ER**	**FUR**	**RP (UN)**^3^
**N**	301	16	320	186	69	11	80	64	71	28	15	14	175	143	126	29	73
**Compliance, aDWT (%)**	58.57	61.54	73.70	42.31	65.83	66.15	59.46	58.00	63.33	64.58	73.47	64.29	68.47	79.35	45.81	85.00	86.42
	58.72			63.65										69.79			
			67.56														
**Mean DWT (h)**	7.92	8.00	9.71	8.59	15.80	8.60	8.85	8.70	9.50	7.75	11.02	9.00	9.92	11.19	10.59	6.80	9.00

HG, headgear; FM, face mask; SP, Schwarz plate; TB, twin-block; PL, Planas appliance; FR, Frankel appliance; Or, other; BIO, Bionator; SA, Sander appliance; ACT, activator; JTB, jumping-the-bite appliance; VBA, van Beek activator; RFA, removable functional appliance; MB, monoblock; CC, cow catch appliance; HR, Hawley plate; ER, Essix retainer; FUR, functional retainer; RP, retainer plate; UN, unspecified; aDWT, actual daily wear time; DWT, daily wear time.

1 group from Tsomos et al study 7.

2 groups from Schott et al, 9 Schott et al, 20 and Zinad et al 21 studies.

3 groups from Tsomos et al7 and Schott et al 20 studies.

Regardless of the type of extra- or intraoral appliance, mean DWT was shorter than recommended, although patients using intraoral appliances cooperated more, as shown by the longer aDWT found in this group (Table 2).

#### DWT vs patient age

The influence of patient age on DWT was assessed only in 17 studies. In 10 of them,^7^^,^^8^^,^^16^, ^17^, ^18^^,^^20^^,^^21^^,^^25^^,^^29^^,^^31^ mean DWT was found to decrease with patient age, whilst in the remaining 7,^9^^,^^23^^,^^26^^,^^28^^,^^36^^,^^39^^,^^40^ no such relationship was found. Schott et al^22^ found that mean DWT is closest to doctors’ recommendations in boys aged 11 to 13 years, and it is shortened both below and above this age range.

#### DWT vs patient sex

The influence of patient sex on DWT was assessed in 20 studies. Sex determined compliance with orthodontist recommendations regarding DWT in 6 studies,^8^^,^^22^^,^^23^^,^^25^^,^^29^^,^^41^ whilst in 12 papers^7^^,^^16^^,^^18^^,^^20^^,^^21^^,^^26^^,^^28^^,^^31^^,^^36^^,^^37^^,^^39^^,^^40^ it was not found to be a significant factor affecting mean DWT.

## Discussion

In the opinion of many authors,^8^^,^^17^^,^^37^ good patient compliance with the recommended DWT during treatment with removable appliances is associated with their ease of use, low failure rate, and lack of discomfort. The results of our study, on the other hand, show that better compliance (aDWT = 86.42%) may also be caused by earlier treatment with fixed appliances. The patients are more aware of the sense of treatment with retainers as they want to maintain the achieved effect. According to some of the authors,^17^ extraoral appliances are worn less willingly than intraoral ones, which is also confirmed by the results of our review (aDWT = 58.72%). This suggests the need for further development of other methods of skeletal anchorage.

In early treatment, different types of appliances are used to prevent malocclusion worsening (interceptive treatment) or to control growth of the maxilla and/or mandible in order to reduce skeletal malocclusion. The larger the size of study groups, the more reliable the assessment of the effectiveness of the appliances. In the articles included in our review, some of the appliances are described in great detail, with their performance documented in extensive studies,^17^^,^^37^^,^^42^ whilst other are presented only as single case reports.^38^^,^^40^ Therefore, it can only be concluded that our review provided reliable results with regard to the cooperation of patients using retainer plates (n = 340, aDWT = 69.79%), Schwarz appliances (n = 320, aDWT = 73.30%), and headgear appliances (n = 301, aDWT = 58.57%). The least reliable data were obtained for the use of a Frankel II appliance (n = 11, aDWT = 66.15%) and face mask (n = 19, aDWT = 61.54%), whilst the aDWT (64.16 %) of patients using functional appliances, that is, those representing the largest study group (n = 713), should be considered evidence-based and reliable.

The issue of patient compliance during treatment with removable appliances has been analysed in several systematic reviews,^43^, ^44^, ^45^ but their authors focussed primarily on determining the relative difference between the objectively measured time and the time recommended by the orthodontist and the time declared by the patient. In addition, it was assessed whether it was important to inform the patient about the presence of the microsensor in the appliance before the beginning of the study. However, none of the studies thoroughly analysed the dependence of patients’ compliance on the type of appliance.

Cozza et al assessed the effectiveness of treatment with intraoral removable appliances and presented the results in a meta-analysis.^46^ However, the studies included did not measure DWT with sensors, so the time recommended by orthodontists was taken into account and at least average patient compliance was assumed. Meanwhile, the results of our review, in which we analysed the aDWT of the most widely represented removable appliances, made it possible to both accurately determine the degree of compliance and identify the types of appliance that are best tolerated by the patient. It appears that these are single-jaw appliances, primarily retainers—functional retainers (aDWT = 85%) and Hawley retainers (aDWT = 79.35%)—although patients using Schwarz active appliances also cooperate well (aDWT = 73.70%). The least acceptable appliance in this category is the Essix retainer, which is worn for far too short a time in relation to the recommendation (aDWT = 45.81%). With regards to activators, rather surprisingly, patients treated with the monoblock appliance are more compliant (aDWT = 73.47%) than those treated with twin-block appliances, which is apparently more convenient as it consists of 2 separate plates (aDWT = 42.31%).

On the other hand, the aDWT analysis performed after the removable appliances used in the studies and included in our review were divided into extraoral, mechanical intraoral (Schwarz appliance), functional intraoral, and removable retainer appliances showed that patients using active appliances cooperated best (aDWT = 73.70%), whilst those wearing retainers were less compliant (aDWT = 69.79%). Patients treated with extraoral appliances (aDWT = 58.72%) were the least compliant with doctors’ recommendations regarding DWT, compared to patients wearing intraoral appliances (aDWT = 67.56%). The excellent compliance of patients wearing retainers may be due to the relatively short recommended wear time and, as mentioned earlier in the discussion, to the greater awareness of patients of the benefits of retainers, as patients are keen to maintain the achieved treatment effect. Schwarz appliances, on the other hand, are widely accepted by patients due to their comfort: They are of simple design compared to many other removable appliances, do not interfere with speech, do not take up much space in the mouth, and do not force specific position of the jaw.

It should be noted here that the calculated ratio of recommended DWT to actual DWT is directly related to the DWT recommended by the orthodontist, and those recommendations are not uniform for each type of appliance. In our review, the longest DWT was reported for Planas appliances (15.80 h/d), followed by Hawley appliances (13.30 h/d) and plate retainers (11.41 h/d). The shortest DWT was observed in patients wearing functional retainers (6.80 h/d) and van Beek activators (7.75 h/d) as well as face masks (8.0 h/d) and headgear (8.05 h/d). Comparison of aDWT results supports the conclusion that the exceptionally good patient compliance regarding the functional retainer (aDWT = 85.00%) with the shortest recorded DWT results from the relatively short recommended wear time (8 h/d). In addition, the small group of patients in whom this appliance was used (n = 29) may reduce the reliability of the results obtained.

The surprisingly low aDWT of the twin-block appliance compared to other intraoral appliances is worthy of in-depth analysis. It is noteworthy that the percentage of this particular appliance in the group treated with functional removable appliances is significantly higher than others, which is reflected in the higher reliability of aDWT than in the other groups. On the other hand, it is quite likely that if the studies were carried out with other appliances, but in larger groups of patients, the aDWT results would approach the low values obtained with the twin-block appliance. It means that the result of the reliable aDWT of twin-block appliances makes it possible to predict, even at the stage of diagnosis, that the compliance of patients wearing this device will be significantly poorer than assumed by clinicians. Such results may discourage the use of functional appliances in treatment. Nevertheless, it should also be borne in mind that DWT required to achieve a clinical effect and thus treat malocclusion may be shorter than recommended by the orthodontist. This was demonstrated in the study by Sarul et al,^13^ who found that with a DWT of 7.5 h, which is close to half the recommended wear time, 80% of patients could be expected to experience complete class II correction.

Despite the confirmed effectiveness of various types of removable orthodontic appliances in the treatment of malocclusion,^47^, ^48^, ^49^ there are still few data in the literature comparing that clinical effect to objectively verified, and therefore reliable, DWT values. Amongst the articles analysed, only 2^13^^,^^17^ provide such data, so this aspect undoubtedly requires further research. Similarly, the contradictory results available in the literature regarding the relationship of patient cooperation to their age and sex should be verified. The current results suggest that such a relationship, even if present, is not clinically relevant to the general population.

## Limitations

The awareness of the presence of sensors can have a positive impact on patient compliance. Some authors have analysed this issue^7^^,^^12^^,^^31^^,^^33^^,^^35^^,^^39^ and, so far, opinions are divided. Nevertheless, it is advisable to take this into account and clearly signal in the research protocol whether the patients were aware of the wear time measurement when wearing appliances.

Another limitation is different follow-up time of patients, which may affect the reliability of the results. This is especially due to the fact that patients may be more willing to comply at the beginning of treatment, but their cooperation wanes over time.

The considerable heterogeneity of papers in terms of age, sex of patients, and follow-up time significantly affects the reliability of the results, further preventing any meta-analysis and drawing more reliable conclusions.

## Conclusions

• Since the best cooperation is only to be expected in the case of intraoral appliances, namely Schwarz appliances, it can be concluded that in the case of other removable appliances an alternative plan has to be created at the beginning of treatment. This applies both to removable activators and in particular to extraoral appliances.
• For satisfactory cooperation, the Essix retainer should not be used in retention treatment.
• The studies that have not reliably assessed patient compliance in relation to treatment efficacy ought to be repeated, as they may significantly verify current DWT recommendations.

## Author contributions

MN collected the data and was the major contributor in writing the manuscript. MS and JL analysed and interpreted the data. MN created the tables. MS gave an idea and the topic. MS and JL performed the revision and final approval of the article.

## Conflict of Interest

None disclosed.

## Appendix Supplementary materials

Image, application 1

Image, application 2

Image, application 3

Image, application 4

## Footnotes

Supplementary material associated with this article can be found in the online version at doi:10.1016/j.identj.2022.07.004.
