# Supplementary material for: Orthodontic Compliance Assessment: A Systematic Review
Source: Int Dent J. 2022 Aug 10;72(5):597–606. doi: 10.1016/j.identj.2022.07.004 (PMC9485511; doi:10.1016/j.identj.2022.07.004)
Supplement: Supplementary file 1 [file mmc1.docx]

Studies included in quantitative synthesis (meta-analysis)
(n = 0)

Studies included in qualitative synthesis
(n = 31)

Full-text articles analyzed for inclusion
(n = 31)

Records screened
(n = 50)

Records after duplicates removed
(n =440)

Additional records identified through other sources
(n = 5)

## Identification

## Eligibility

## Included

## Screening

Records identified through database searching
(n =451)

Appendix Figure 1. Flow chart

Full-text articles excluded
(n = 19)

Records excluded
(n = 390)
