# Supplementary material for: Orthodontic Compliance Assessment: A Systematic Review
Source: Int Dent J. 2022 Aug 10;72(5):597–606. doi: 10.1016/j.identj.2022.07.004 (PMC9485511; doi:10.1016/j.identj.2022.07.004)
Supplement: Supplementary file 2 [file mmc2.docx]

**Appendix Table 1. Search strategy and findings per database**

| **Database** | **Search strategy** | **Findings** |
| --- | --- | --- |
| **Embase** | #1 AND #2 | 50 |
|  | #1 ('removable orthodontic appliances' OR 'removable appliances' OR 'orthodontic appliances' OR 'removable oral appliances' OR 'functional orthodontic appliances' OR 'orthodontic retainers') AND [embase]/lim | 4,005 |
|  | #2 (microsensor OR sensor OR "electronic timer" OR "microelectronic wear-time device" OR "microelectronic sensor" OR "wear-time documentation" OR "electronic wear-time measurements" OR "microelectronic wear-time documentation") AND [embase]/lim | 146,140 |
| **Pubmed** | #1 AND #2 | 74 |
|  | #1 [All Fields] ("Removable orthodontic appliances" OR "removable appliances" OR "orthodontic appliances" OR "removable oral appliances" OR "functional orthodontic appliances" OR "orthodontic retainers") | 16,061 |
|  | #2 [All Fields] (microsensor OR sensor OR "electronic timer" OR "microelectronic wear-time device" OR "microelectronic sensor" OR "wear-time documentation" OR "electronic wear-time measurements" OR "microelectronic wear-time documentation") | 199,357 |
| **Scopus** | #1 AND #2 | 376 |
|  | #1 ALL ("Removable orthodontic appliances" OR "removable appliances" OR "orthodontic appliances" OR "removable oral appliances" OR "functional orthodontic appliances" OR "orthodontic retainers") | 25,735 |
|  | #2 ALL (microsensor OR sensor OR "electronic timer" OR "microelectronic weartime device" OR "microelectronic sensor" OR "wear-time documentation" OR "electronic wear-time measurements" OR "microelectronic wear-time documentation") | 3,390,856 |
| **Web of Science** | #1 AND #2 | 42 |
|  | #1 ALL= ("Removable orthodontic appliances" OR "removable appliances" OR "orthodontic appliances" OR "removable oral appliances" OR "functional orthodontic appliances" OR "orthodontic retainers") | 2,395 |
|  | #2 ALL= (microsensor OR sensor OR "electronic timer" OR "microelectronic wear-time device" OR "microelectronic sensor" OR "wear-time documentation" OR "electronic wear-time measurements" OR "microelectronic wear-time documentation") | 1,051,868 |
