# Supplementary material for: Orthodontic Compliance Assessment: A Systematic Review
Source: Int Dent J. 2022 Aug 10;72(5):597–606. doi: 10.1016/j.identj.2022.07.004 (PMC9485511; doi:10.1016/j.identj.2022.07.004)
Supplement: Supplementary file 3 [file mmc3.docx]

**Appendix Table 2.** Rationale for exclusion of the articles

| **Study** | **Title of the article** | **Reason of exclusion** |
| --- | --- | --- |
| Witt et al., 1991 | Tragezeitmesser in der KieferorthopS.dieee  cui bono? Uberlegungen zum State-of-the-art  bei Tragezeitmessung und Complianceforschung  in der Kieferorthopfidie | Subjective study based  entirely on a questionnaire |
| Witt et al., 1992 | Tragezeitverordnungen bei herausnehmbaren  Gerfiten- Ergebnisse einer Umfrage | Subjective study based  entirely on a questionnaire |
| Schott et al.,  2009 | Young patients’ attitudes toward removable  appliance wear times, wear-time instructions  and electronic wear-time measurements - Results of a  questionnaire study | Subjective study based  entirely on a questionnaire |
| Schott et al.,  2010 | Applicative characteristics of new microelectronic  sensors Smart Retainer® and TheraMon® for  measuring wear time | In-vitro study |
| Schott et al.,  2011a | Color fading of the blue compliance indicator  encapsulated in removable clear Invisalign Teen®  aligners | Small sample  Sensor doesn’t measure  objectively the average of  daily wear time (DWT) |
| Schott et al.,  2011b | Wearing times of orthodontic devices as measured by  the TheraMon® microsensor | Only a description of a  single case in the sample |
| Vanderveken et al., 2012 | Objective measurement of compliance during oral  appliance therapy for sleep-disordered breathing | Different treatment protocol (mandibular advancement device for SDB) |
| Dieltjens et al., 2013 | Objectively Measured vs Self-Reported  Compliance During Oral Appliance  Therapy for Sleep-Disordered Breathing | Different treatment protocol (mandibular advancement device for SDB) |
| Pauls et al., 2013 | Effects of wear time recording on the patient’s compliance. | Unspecified appliance type (Schwarz appliances, activators, jumping-the-bite appliances) |
| Schott et al., 2013 | Questionnaire study of electronic wear-time tracking  as experienced by patients and parents during treatment with removable orthodontic appliances | Subjective study based  entirely on a questionnaire |
| Schott TC and Ludwig B., 2014 | Microelectronic wear-time documentation of removable orthodontic devices detects heterogeneous wear behavior and individualizes treatment planning. | Unspecified appliance type (Schwarz appliances and functional appliances) |
| Veeroo et al., 2014 | Motivation and compliance with intraoral elastics | Different treatment protocol (patients with fixed appliances) |
| Sarul et al., 2016 | Objectification of Orthodontic Treatment Needs: Does the Classification of Malocclusions or a History of Orthodontic Treatment Matter? | DWT assessed according to severity of malocclusion (and not the type of appliance) |
| Brierley et  al., 2017 | How accurate are TheraMon® microsensors at  measuring intraoral wear time? Recorded vs. actual  wear times in five volunteers | Small sample  Low observation time  Sensor in fixed appliances |
| Sarul et al.,  2017 | Objectively measured patient cooperation during early  orthodontic treatment. Does psychology have an  impact? | Subjective study based  entirely on a questionnaire |
| Sarul et al., 2017 | Objectively measured compliance during early orthodontic treatment: Do treatment needs have an impact? | Unspecified appliance type (Schwarz appliances and Twin-Blocks) |
| El-Huni et  al., 2018 | Understanding factors influencing compliance with  removable functional appliances. A qualitative study | Subjective study based  entirely on a questionnaire |
| Kirshenblatt  et al., 2018 | Adherence to treatment with removable oral appliances:  the past and the future | Microsensor review |
| Schott et al., 2018 | How patient-selected colors for removable appliances are reflected in  electronically tracked compliance (wear times and wear behavior) | Classification of the appliances according to colours (and not the appliance type) |
