# Supplementary material for: Orthodontic Compliance Assessment: A Systematic Review
Source: Int Dent J. 2022 Aug 10;72(5):597–606. doi: 10.1016/j.identj.2022.07.004 (PMC9485511; doi:10.1016/j.identj.2022.07.004)
Supplement: Supplementary file 4 [file mmc4.docx]

**Appendix Table 3.** Cochrane risk of bias tool for the randomized clinical trials

| **Study** | **Random sequence generation** | **Allocation concealment** | **Blinding of participants and personnel** | **Blinding of an outcome assessment** | **Incomplete outcome data** | **Selective reporting** | **Other bias** |
| --- | --- | --- | --- | --- | --- | --- | --- |
| Ackerman et al., 2011 [12] | Unclear | Low | Low | Unclear | Low | Unclear | Unclear |
| Hyun et al., 2015 [38] | Low | Low | Low | Unclear | Low | Unclear | Unclear |
| Parekh et al., 2018 [40] | Low | Low | Low | Low | Low | Low | Low |
| Al-Moghrabi et al., 2019 [37] | Low | Low | Low | Low | Low | Unclear | Low |
| Vagdouti et al., 2019 [39] | Low | Low | Low | Low | Low | Unclear | Low |

**Appendix Table 4.** Newcastle-Ottawa Scale for assessing the quality of cohort studies

| STUDY | **SELECTION (max 4 points)** | | | | | | | **COMPARABILITY (max 2 points)** | **OUTCOME (max 3 points)** | | | **SCORE (out of 9)** |
| --- | --- | --- | --- | --- | --- | --- | --- | --- | --- | --- | --- | --- |
|  | Representat  iveness of  the exposed  cohort | | Selection  of the  nonexposed  cohort | | Ascertain  ment of  exposure | | Demonstration  that outcome of  interest was not  present at start  of study | Comparability of  cohorts on the basis of  the design or analysis | Assessment  of outcome | Was  follow-up  long  enough for  outcomes  to occur? | Adequacy of the follow-up  of  cohorts |  |
| Clemmer et al., 1979 [34] | 1 | |  | |  | | 1 | 2 | 1 | 1 | 1 | 7 |
| Cureton et al., 1993 [16] | 1 | |  | | 1 | | 1 | 2 | 1 | 1 | 1 | 8 |
| Cureton et al., 1993 [28] | 1 | |  | |  | | 1 | 2 | 1 | 1 | 1 | 7 |
| Barstch et al., 1993 [17] | 1 | |  | | 1 | | 1 | 2 | 1 | 1 | 1 | 8 |
| Cole et al., 2002 [18] | 1 | |  | | 1 | | 1 | 2 | 1 | 1 | 1 | 8 |
| Agar et al., 2005 [19] | 1 | |  | | 1 | | 1 | 2 | 1 | 1 | 1 | 8 |
| Brandao et al., 2006 [20] | 1 | |  | | 1 | | 1 | 2 | 1 | 1 | 1 | 8 |
| Bos et al.,  2007 [29] | 1 | |  | |  | | 1 | 2 | 1 | 1 | 1 | 7 |
| Trakyali et al., 2008 [21] | 1 | |  | | 1 | | 1 | 2 | 1 | 1 | 1 | 8 |
| Kawala et  al., 2013 [22] | 1 |  | | 1 | | 1 | | 2 | 1 | 1 | 1 | 8 |
| Schott et  al., 2013 [6] | 1 |  | |  | | 1 | | 2 | 1 | 1 | 1 | 7 |
| Schäfer et  al., 2014 [8] | 1 |  | | 1 | | 1 | | 2 | 1 | 1 | 1 | 8 |
| Schott et  al., 2014 [9] | 1 |  | |  | | 1 | | 2 | 1 | 1 | 1 | 7 |
| Schott et  al., 2014 [30] | 1 |  | | 1 | | 1 | | 2 | 1 | 1 | 1 | 8 |
| Tsomos et  al, 2014 [7] | 1 |  | | 1 | | 1 | | 2 | 1 | 1 | 1 | 8 |
| Al-Kurwi et  al., 2016 [23] | 1 |  | | 1 | | 1 | | 2 | 1 | 1 | 1 | 8 |
| Arreghini et  al., 2016 [24] | 1 |  | | 1 | | 1 | | 2 | 1 | 1 | 1 | 8 |
| Schott et  al., 2017 [25] | 1 |  | | 1 | | 1 | | 2 | 1 | 1 | 1 | 8 |
| Zinad et al.,  2017 [26] | 1 |  | | 1 | | 1 | | 2 | 1 | 1 | 1 | 8 |
| Charavet et  al., 2018 [31] | 1 |  | |  | | 1 | | 2 | 1 | 1 | 1 | 7 |
| Von  Bremen et  al., 2018 [32] | 1 |  | |  | | 1 | | 2 | 1 | 1 | 1 | 7 |
| Huanca et  al., 2019 [27] | 1 |  | | 1 | | 1 | | 2 | 1 | 1 | 1 | 8 |
| Sarul et al., 2019 [35] | 1 |  | |  | | 1 | | 2 | 1 | 1 | 1 | 7 |
| Arponen et  al., 2020 [33] | 1 |  | |  | | 1 | | 2 | 1 | 1 | 1 | 7 |
| Kutay et al., 2021 [15] | 1 |  | | 1 | | 1 | | 2 | 1 | 1 | 1 | 8 |
| Sarul et al., 2021 [13] | 1 |  | | 1 | | 1 | | 2 | 1 | 1 | 1 | 8 |
